# Urban nexus and transformative pathways towards resilient cities: A case of the Gauteng City-Region, South Africa

**DOI:** 10.1016/j.cities.2021.103266

**Published:** 2021-05-23

**Authors:** Luxon Nhamo, Lameck Rwizi, Sylvester Mpandeli, Joel Botai, James Magidi, Henerica Tazvinga, Nafiisa Sobratee, Stanley Liphadzi, Dhesigen Naidoo, Albert T. Modi, Rob Slotow, Tafadzwanashe Mabhaudhi

**Affiliations:** a Water Research Commission of South Africa (WRC), Lynnwood Manor, Pretoria 0081, South Africa; b College of Agriculture and Environmental Sciences (CAES), University of South Africa (UNISA), Florida, Johannesburg 1710, South Africa; c South Africa Weather Services (SAWS), Ecoglades, Centurion 0157, Pretoria, South Africa; d Geomatics Department, Tshwane University of Technology, Pretoria, 0001, South Africa; e School of Life Sciences, University of KwaZulu-Natal, Scottsville, Pietermaritzburg 3209, South Africa; f Centre for Transformative Agricultural and Food Systems (CTAFS), School of Agricultural, Earth and Environmental Sciences, University of KwaZulu-Natal, Scottsville, Pietermaritzburg 3209, South Africa; g School of Environmental Sciences, University of Venda, Thohoyandou 0950, South Africa; h Department of Genetics, Evolution and Environment, University College London, WC1E 6BT, United Kingdom; i Centre for Water Resources Research (CWRR), School of Agricultural, Earth and Environmental Sciences, University of KwaZulu-Natal, Scottsville, Pietermaritzburg 3209, South Africa

**Keywords:** Adaptation, circular economy, migration, sustainability, urbanisation, urban planning

## Abstract

Challenges emanating from rapid urbanisation require innovative strategies to transform cities into global climate action and adaptation centres. We provide an analysis of the impacts of rapid urbanisation in the Gauteng City-Region, South Africa, highlighting major challenges related to (i) land use management, (ii) service delivery (water, energy, food, and waste and sanitation), and (iii) social cohesion. Geospatial techniques were used to assess spatio-temporal changes in the urban landscapes, including variations in land surface temperatures. Massive impervious surfaces, rising temperatures, flooding and heatwaves are exacerbating the challenges associated with rapid urbanisation. An outline of the response pathways towards sustainable and resilient cities is given as a lens to formulate informed and coherent adaptation urban planning strategies. The assessment facilitated developing a contextualised conceptual framework, focusing on demographic, climatic, and environmental changes, and the risks associated with rapid urbanisation. If not well managed in an integrated manner, rapid urbanisation poses a huge environmental and human health risk and could retard progress towards sustainable cities by 2030. Nexus planning provides the lens and basis to achieve urban resilience, by integrating complex, but interlinked sectors, by considering both ecological and built infrastructures, in a balanced manner, as key to resilience and adaptation strategies.

## 1 Introduction

Although urban areas provide economic growth and human development opportunities, they are also a source of anthropogenic environmental impacts, as they consume more than 70% of global energy and materials (Avtar et al., 2019; Riffat et al., 2016). Coupled with climate change, rapid urbanisation contributes to the insecurity, depletion, and degradation, of the existing resource base (Beard et al., 2016; UNGA, 2015). The challenges are more pronounced in the developing world, where cities struggle to cope with the influx of people, as demand for resources often outstrips supply (Collier and Venables, 2017). Cities in developing countries generally fail to provide clean water and sanitation, sufficient food, or clean and affordable energy to a rapidly urbanising population (Dos Santos et al., 2017; Simatele and Simatele, 2015). In the case of sub-Saharan Africa (SSA), the projected population increase to about 2 billion people by 2050, of which 60% will be living in cities, will only exacerbate the challenges associated with rapid urbanisation, such as pollution, poor waste management, resource insecurity, urban sprawl, and high unemployment and crime rates (Van Bavel, 2013). Efforts should be directed towards building urban resilience, including transforming urban areas into centres for climate action (Mpandeli et al., 2018; Nhamo et al., 2018). This is based on the Intergovernmental Panel on Climate Change’s (IPCC) findings that identified cities as important sites of global climate action and adaptation (Hoegh-Guldberg et al., 2018).

The challenges associated with rapid urbanisation are complex and cross-cutting. Therefore, they require polycentric and transformative approaches to achieve urban sustainability, particularly considering that urban areas are complex systems superimposed on the natural environment. Of concern at the global level, by 2030 urban growth is projected to encroach into protected areas by more than three times from the 2000 base area of 450,000 km^2^. The encroachment into biodiversity hotspots (areas with high concentrations of endemic species facing development threats) will quadruple during the same period (Güneralp et al., 2013). In SSA alone, urban encroachment into ecological infrastructure will increase by more than 20% during the same period (Güneralp et al., 2013). Rapid urbanisation and climate change, together with unsustainable land management practices, are reducing agricultural land, degrading ecological systems, and modifying landscapes and biodiversity, putting a strain on the provision of essential services like waste management and sanitation (McKinney, 2008; Satterthwaite et al., 2010). The challenges are significantly impacting food systems, industrial production, and urban life (Satterthwaite et al., 2010). The need to adapt cities to the difficulties associated with rapid urbanisation, and to accommodate economic and climate refugees, culminated in the formulation, in 2015, of the Sustainable Development Goal (SDG) 11, which commit to “make cities and human settlements inclusive, safe, resilient and sustainable by 2030” (UNGA, 2015). Achieving this goal requires enhancing urban preparedness for future socio-ecological changes.

Transformative approaches, such as nexus planning and circular economy, are poised to expedite urban resilience and sustainability (Hettiarachchi and Ardakanian, 2016; Nhamo et al., 2020). These approaches are suited for sustainability planning, as they enhance resource recycling, regeneration, management, and resource security (Lehmann, 2018; Martin and Fischer, 2012; Sperling and Berke, 2017). Sustainability focused approaches complement each other. For example, nexus planning and circular economy both form an integral part of urban planning and are designed to ensure the most efficient use and management of resources (Lehmann, 2018; Satterthwaite et al., 2010). Furthermore, nexus planning is a source for informed policies, and is essential for planning and developing management decisions for the circular economy (Lehmann, 2018). Nexus planning also guides scenario planning decisions in urban planning by identifying priority areas for intervention (Johnson and Karlberg, 2017; Star et al., 2016). Both nexus planning and scenario planning have the advantage of providing alternatives that cover key uncertainties, providing the basis for discussions about strategic policy formulations, the relative effectiveness of various management options, innovation, and community visions (Granderson, 2014). Therefore, nexus planning, circular economy and scenario planning are important pathways towards resilient cities.

Current linear approaches in urban planning only aggravate contemporary crises associated with rapid urbanisation and exacerbate socio-economic inequalities (Nhamo and Ndlela, 2020). A nexus of transformative approaches (nexus planning, circular economy, and scenario planning) in urban planning provides pathways to manage trade-offs and synergies in an integrated manner, opening new possibilities for practice (Albrechts et al., 2020). The term “urban nexus” represents the relations, interconnectedness, and interdependencies in urban systems (energy, water, and food and material provisioning systems) and the need for integrated, transformative approaches across these sectors (Lehmann, 2018). It emphasises integrating resource management processes that increase resource use efficiency and smart infrastructural systems, transforms urban planning practice, and reduce gas emissions and waste generation, thus linking the three transformative processes. Urban nexus does not only focus on the present situation but also consider inter-generational sustainability. This is based on an approach that the future has to be imagined as radically and structurally different from the present situation (Pereira et al., 2018).

Such planning is long overdue for the Gauteng City-Region, which has a diverse immigrant population and is witnessing unprecedented growth and inequality (Baffi et al., 2018; Todes and Turok, 2018). These challenges pose the risk of resource insecurity and conflict in the absence of transformative strategies, and overcrowding poses a considerable risk to human health and sanitation (Wolf, 2007).

The urban nexus concept is essential for informing decision-making on strategic policy formulations related to socio, climatic, environmental changes, and their impacts on urban landscapes. We developed a contextualised adaptation framework and pathways towards urban resilience, taking the Gauteng City-Region as a case study. The framework integrates the three main elements of sustainability, mainly ecological, economic, and social elements, holistically, and provide a platform for implementing transformative approaches towards sustainable cities, by linking urban nexus with urban planning.

## 2 Methods

### 2.1 Description of the study area

**Fig. 1:**
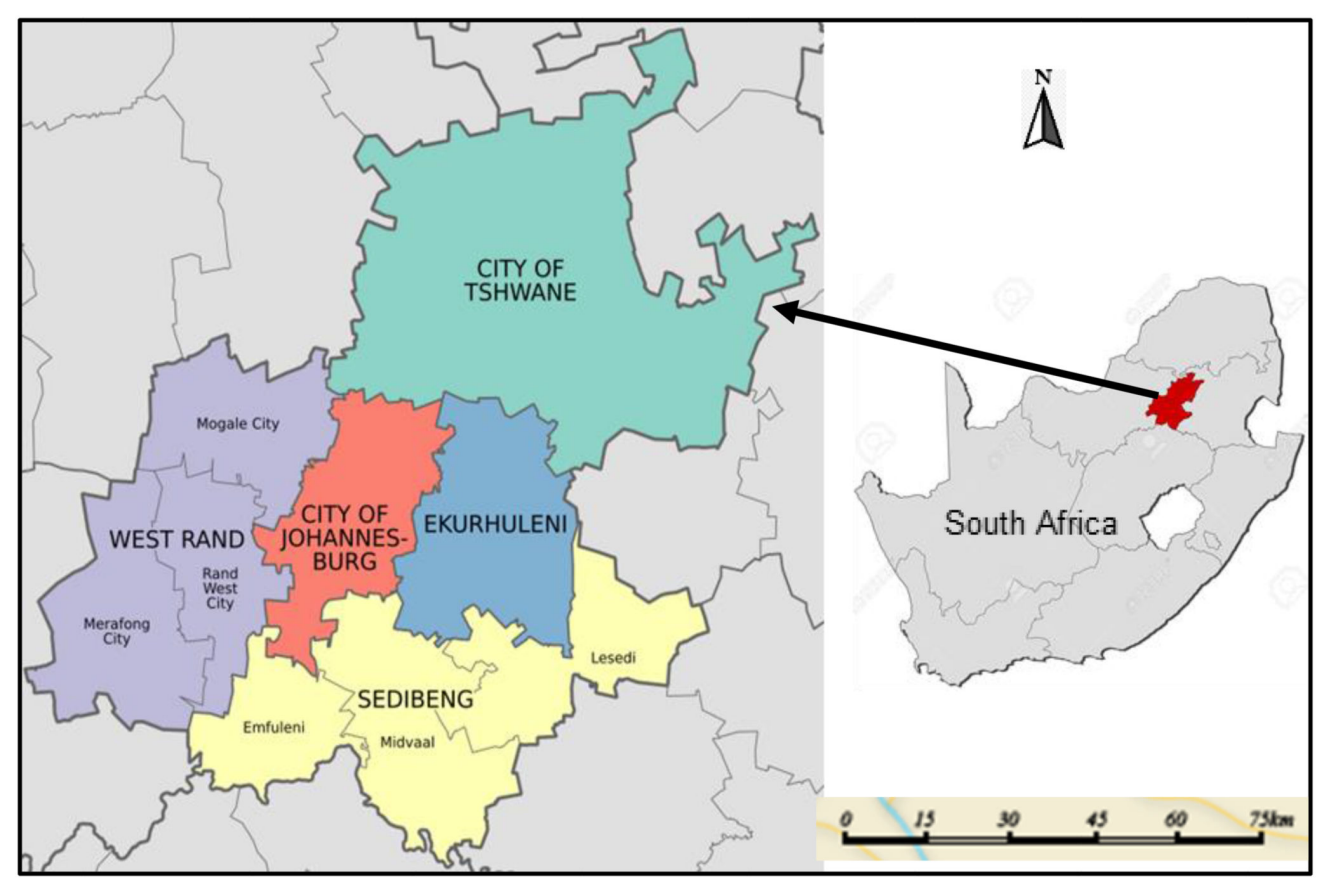
The Gauteng Province showing the metropolitan areas, which include Johannesburg, Tshwane, and Ekurhuleni, as well as the two district municipalities of West Rand and Sedibeng, with their component local municipalities.

The Gauteng City-Region (Fig. 1) is an integrated cluster of urban areas that form the Gauteng Province, an urbanised province and economic heartland of South Africa. The metropolitan areas that together make up the province include Johannesburg (South Africa’s largest city), Tshwane (the capital city and formerly called Pretoria), Ekurhuleni, as well as industrial and mining centres of Germiston, Springs, Alberton, Boksburg, Benoni, Vereeniging, Vanderbijlpark, Krugersdorp, Randfontein, and Westonaria (Fig. 1).

The industrial and economic development of Gauteng Province has made the province the main centre of trade, commerce, and industrial development of South Africa. It is the largest economic hub of southern Africa and the rest of the African continent (Chakwizira et al., 2018). The province contributes 40% of the national gross domestic product (GDP), making it an attractive destination for migrants (Baffi et al., 2018; Bobbins and Culwick, 2015; StatsSA, 2019). The employment opportunities presented by the Gauteng City-Region due to its industrial and economic development, have contributed to its rapid urbanisation.

Although the province is important to national and regional economies, its total land area is only 18,170 km^2^ (representing only 2% of the total national land area), the smallest of all the nine provinces of South Africa. Besides its small size, it is the most densely populated, with a population of over 15,200,000 people (StatsSA, 2019). Therefore, economic and social opportunities are spatially concentrated in Gauteng, a position that is attractive for migrants (Bobbins and Culwick, 2015; StatsSA, 2019). Due to rapid urbanisation, the province is faced with a host of challenges that include high crime rates, urban sprawl, pollution, poor waste management unemployment, and social exclusion and inequalities (Baffi et al., 2018). The spatial distribution of diverse populations in the province is characterised by the high number of informal settlements surrounding highly affluent suburbs (Baffi et al., 2018).

### 2.2 Conceptual framework

**Fig. 2:**
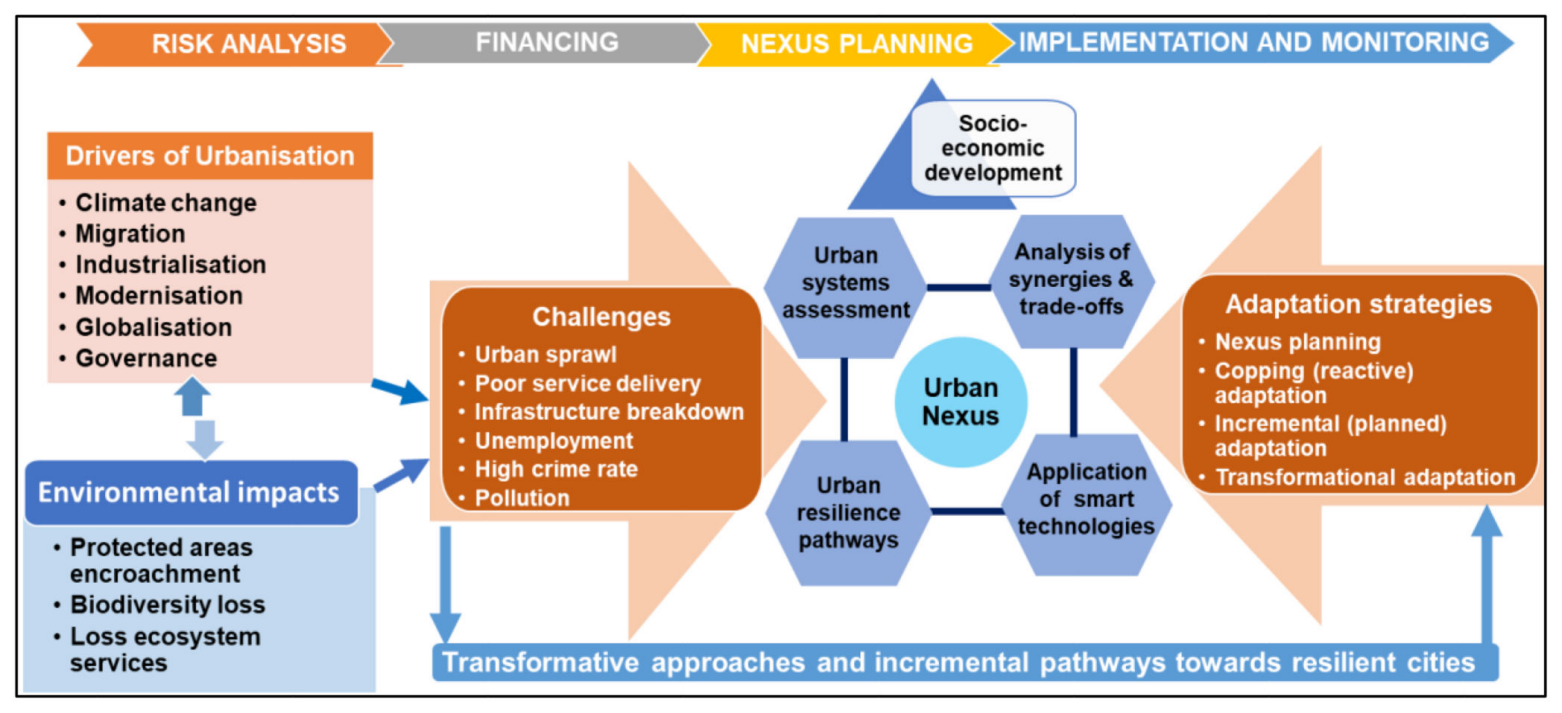
An urban nexus methodological framework showing process and pathways towards resilient cities and linking the urban nexus approach with sustainability.

There are four main thematic areas needed to understand urban dynamics and processes that can transform urban spaces into centres of climate action and human development, including risk analysis, financing, nexus planning, and implementation and monitoring (Fig. 2). Risk analysis involves exploring urbanisation drivers, which include climate change, migration, modernisation, industrialisation, governance, and globalisation. This is necessary for understanding the contribution of the changes in urban landscapes to ecological infrastructure. In the Gauteng City-Region case, the urbanised region faces the challenges of urban sprawl, poor service delivery, infrastructure breakdown, high unemployment and crime rates, and pollution due to rapid population growth and expansion of the built environment.

The nexus planning thematic areas involve the modelling processes of urban dynamics to provide strategic solutions to urban planners that lead to ecologically sustainable economic and social development. Nexus planning forms part of transformative analyses that identify priority interventional areas. The analyses include urban systems assessment, analysis of synergies and trade-offs, application of smart technologies, and build urban resilience pathways (Webb et al., 2018) (Fig. 2). Transformative approaches (nexus planning, circular economy, and scenario planning), together with incremental pathways provide tools that enhance resilience and adapt cities to the anticipated influx of migrants and climate change, by providing strategic, resilient pathways (Lehmann, 2018). However, these interventions’ success requires a financial investment to implement, monitor, and evaluate progress.

The approach builds on the understanding that urban areas are sites of human development, climate action, and adaptation. They have the systems to cope with rapid urbanisation and adapt to change without disturbing their systems (McGranahan et al., 2016). The premise is to transition urban areas towards a circular economy and achieve resilience.

### 2.3 Remote sensing analysis

Remotely sensed products obtained from the National Aeronautics and Space Administration’s (NASA) Earthdata portal were used to assess changes in land surface temperature (LST), and in urban landscapes, from 1986 to 2018, using geospatial techniques. The Moderate Resolution Imaging Spectroradiometer (MODIS) and Landsat products from NASA, provided the primary remotely sensed data to assess these changes over time. The Semi-Automatic Classification Plugin (SCP) (a QGIS tool) was used to convert digital numbers (DN) to Top of Atmosphere (ToA) reflectance. The *R*-programming language was essential for separating built-up areas from the other landuses, using the random forest machine learning classifier (Chen et al., 2018; Stepinac and Gašparović, 2020). Classified images were sieved to eliminate small and insignificant areas, and the landscape metrics were quantified in the built-up area for each of the respective years under review.

The LST was extracted from the MODIS LST product (MOD11A2), with an 8-day temporal resolution and 1 km spatial resolution. The LST analysis covered the period between February 2000 and February 2019 to consider the spatio-temporal variations over time. The algorithm to retrieve the LST was run on Google Earth Engine (GEE) from Google.

Atmospheric nitrogen dioxide (NO_2_) was obtained from the Global Ozone Monitoring Experiment (GOME) satellite instrument (Burrows et al., 1999), which also collects data on other various atmospheric gases such as ozone (O_3_), methane (CH_4_), formaldehyde, (HCHO), carbon monoxide (CO), and sulphur dioxide (SO_2_) (Omrani et al., 2020; Theys et al., 2017). The Copernicus Program’s TROPOMI (TROPOspheric Monitoring Instrument) captures the density of various atmospheric gases, aerosols, and clouds (Theys et al., 2017). These data are available from the Copernicus Open Access Data Hub and were accessed through the Google Earth Engine (GEE), a cloud -computing platform for storing spatial datasets (Gorelick et al., 2017).

## 3 Results

### 3.2 Environmental changes in Gauteng Province

#### 3.2.1 Increases in the built-up area and land surface temperatures (LST)

**Fig. 3:**
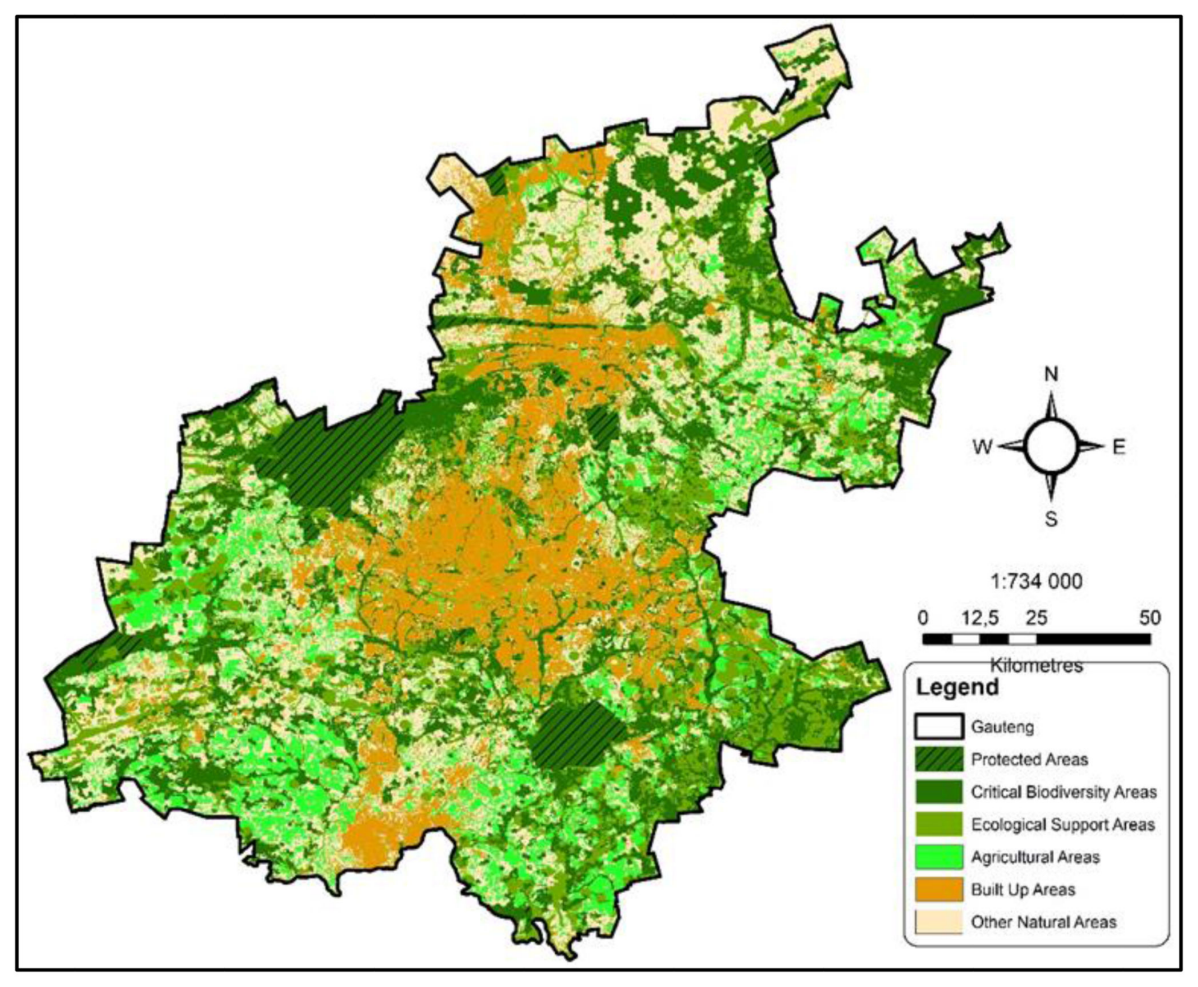
The biodiversity sector plan map for Gauteng Province. The Critical Biodiversity Areas and Ecological Support Areas were mapped in 2011 (Compaan, 2011), and the agricultural and built-up areas (transformational layer) were mapped in 2020.

Rapid urbanisation is evidenced by the considerable gains in the built environment in Gauteng between 1986 and 2019 (Table 1). The proportion of built-up area (or built environment) against other landuses between 1986 and 2019 increased by 62.5% (Table 1). The built environment only occupied 7,83% of the total provincial land area in 1986, but the proportion increased to 21,19% in 2019. Between 2000 and 2019, the population almost doubled from 8,8 million to 15,1 million, hence the significant changes in the province’s urban landscape (Fig. 3).

The significant increase in the built-up area between 1986 and 2019 indicates rapid urbanisation, and projections suggest that population density in the province will double from the current 58 persons per hectare to 105 persons per hectare between 2005 and 2050 (Landau and Gindrey, 2008). At an average annual growth rate of 2% between 2008 and 2058, the province’s population is projected to reach 28 million people (Landau and Gindrey, 2008).

During the same period (2000 to 2019), average annual temperatures increased from about 25°C in 2000 to more than 30°C in 2019, indicating that the LST increased at the same time when the build-up-area was also increasing. Thus, the expansion in the built-up area and the increase in the impervious surface contribute to the increasing LST. Previous studies have established a positive correlation between buildings’ concentration and the vast expanse of impervious surfaces with high temperatures (Hua et al., 2020; Yuan and Bauer, 2007).

#### 3.2.2 Encroachment of built environment into other ecological systems

In 2011, the Gauteng Provincial Government mapped the distribution of biodiversity in the province (Compaan, 2011). We overlaid the province’s current built-up area to understand better the impact of the built environment on the ecological infrastructure (Fig. 3). The zonal statistical analysis produced the results shown in Table 2. The biodiversity distribution map (Fig. 3) includes Critical Biodiversity Areas (CBA) (areas needed to meet biodiversity targets for ecosystems, species, and ecological processes), and Ecological Biodiversity Areas (EBA) (areas that support the ecological functioning of Critical Biodiversity Areas and in delivering ecosystem services). Mapping CBAs and ESAs is essential for guiding decision-making about the most suitable areas to locate developmental areas.

By 2020, agricultural land expansion had reduced the ecological infrastructure by 40.9%, and the growth of the built environment encroached into ecological infrastructure by a further 15.1%. The transformation of 56% of the province’s critical ecological infrastructure in only 10 years is severely compromising progress towards achieving an urban circular economy and sustainable provision of ecosystem services and goods (Colavitti et al., 2020).

**Fig. 4:**
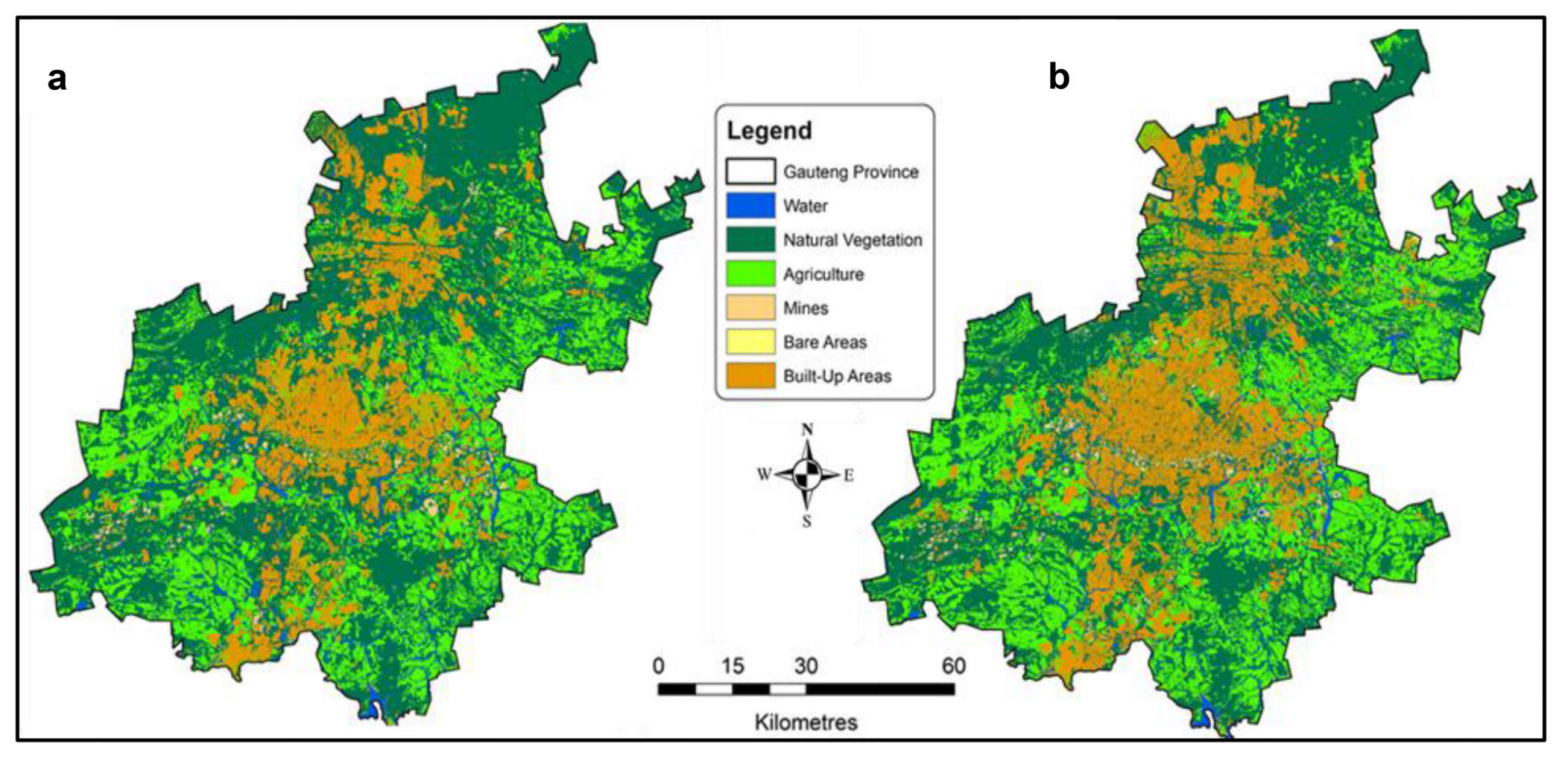
Encroachment of built environment into ecological infrastructure in Gauteng Province between 2010 (map a) and 2018 (map b).

The increase in the built environment, and the subsequent changes in the urban landscape significantly reduced the ecological landscape between 2011 and 2018 (Fig. 4). Waterbodies were reduced by almost 14% and vegetated land by over 10% during the same period (Table 3). The biodiversity map (Fig. 3) was developed in 2011. The 2011 and 2020 maps landuse maps (Fig. 4) were overlaid on the biodiversity map to appreciate the impact of the built environment on ecological infrastructure.

There was a notable increase in cleared land area (about 1 122%), indicating that more natural land or greenfields are being turned into the built environment. The built environment increased by over 23% between 2011 and 2018 (Table 3). Urban growth is, thus, disturbing critical biodiversity areas and other ecological systems, disrupting natural flows, and impacting on ecosystem services, the hydrological system, causing temperature rise, and reductions in urban agriculture, as also identified by previous research (Elmqvist et al., 2016; SANBI, 2018).

#### 3.2.3 Levels of pollution

In terms of air pollution, the Gauteng Province was the most polluting province of all the nine South African provinces. The heavy air pollution levels are compounded by rapid urbanisation and vehicular pollution (Chakwizira et al., 2018). Table 4 indicates the contribution of vehicular pollution and CO_2_ emissions in Gauteng in comparison with national figures. Gauteng has the highest pollutant emission rates for all pollutants in the country due to rapid urbanisation, increased volume of vehicles, and destruction of the ecological infrastructure which plays a vital role in reducing pollution (Hewitt et al., 2020; Kumar et al., 2019).

### 3.3 Urban nexus as a platform to build urban resilience

**Fig. 5:**
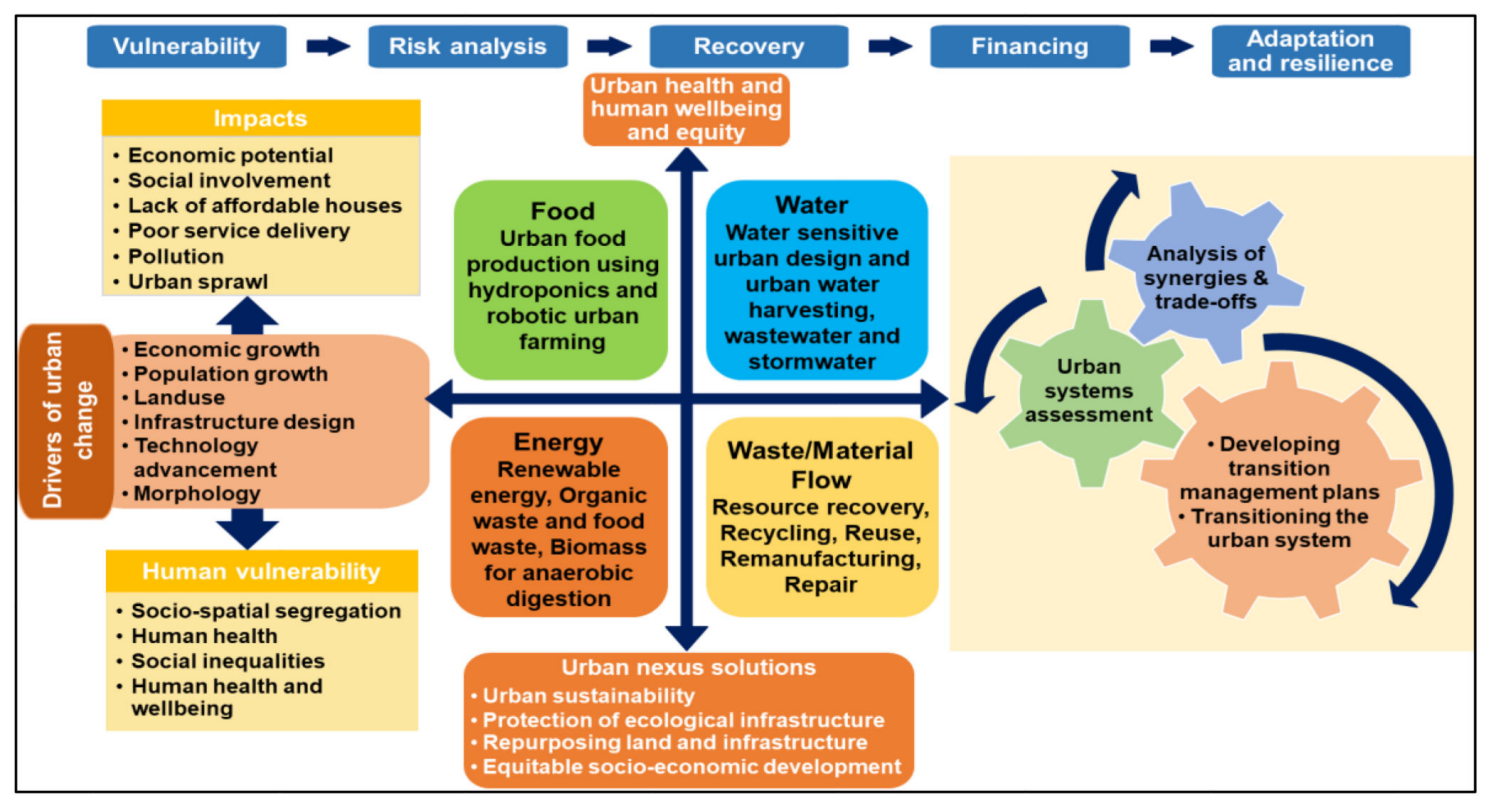
A contextualised urban nexus framework for the Gauteng City-Region showing the processes involved in transformative urban planning.

The challenges brought about by rapid urbanisation in the Gauteng City-Region are multidisciplinary, intertwined, and cross-sectoral (urban sprawl, overpopulation, poor sanitation, encroachment into ecological infrastructure, disturbance of wildlife habitats, the emergence of novel pathogens, poor waste management, water, food, and nutrition insecurity, ageing infrastructure, amongst others), and, thus, require polycentric and transformative approaches that provide objective and integrated assessments (GPG, 2017). The Gauteng Spatial Development Framework (GSDF) acknowledges the need for a balanced polycentric spatial approach to attain a sustainable and resilient Gauteng City-Region. Still, it lacks clear, integrated pathways to achieve the vision for a transformation, modernisation, and re-industrialisation (GPG, 2017). Therefore, this section proposes stepwise transformative pathways to guide the designing and creation of a balanced urban system and integrated management of resources by promoting the reduction of waste in the whole value chain of the economy (Meyer and Auriacombe, 2019; Nhamo et al., 2020). The proposed urban nexus approach is an integral component of sustainable resource management and urban planning. It addresses the broad linkages and interdependencies of urban systems, including energy, water, food, and material provisioning systems. Thus, nexus planning provides the platform to incorporate different but interlinked sectors of a system into an integrated approach for collective outcomes. The essence of the urban nexus approach is to ensure resource use efficiency, sustainable infrastructural systems, transformative planning practices, waste management, and reductions in carbon emissions (Fig. 5). It provides implementation pathways of the provincial spatial infrastructure framework.

A contextualised urban nexus framework for the Gauteng City-Region (Fig. 5) illustrates the implementation processes to build resilience and cope with the rapid urbanisation. The adaptation processes guide policy-makers to formulate coherent strategies that lead to preparedness and resilience. This integrated urban systems assessment links the urban input streams that include urban change drivers, urban metabolism, business pragmatism, environment and resources, urban governance, sustainable development, and security and geopolitics. The urban nexus modelling identifies trade-offs and synergies, inform on the risks, and provides solutions through scenario planning (Nhamo and Ndlela, 2020). This is also possible through smart technologies to enhance adaptation and resilience and financial resources to ensure effective implementation and monitoring. Thus, urban sustainability is achieved by addressing the five thematic areas that include vulnerability, risk analysis, recovery, financing, adaptation, and resilience (Fig. 5).

As the urban landscape is always changing, nexus planning informs policy and decision-making to formulate coherent strategies during urban planning (Sharmina et al., 2016). Unplanned landuse modifications impact essential services and are a risk to environmental and human health (Nhamo and Ndlela, 2020). The urban nexus conceptual framework is designed to drive urban areas towards an urban circular economy and attain urban resilience and cleaner production. The essence is to establish a balance between economic development and environmental and resources protection, emphasising the most efficient use and recycling of resources, and environmental protection (Murray et al., 2017). It differs from a linear economy (make-use-dispose model) in that it is characterised by low energy consumption and emission of pollutants, and high efficiency in resource use through the adoption of cleaner production processes, eco-industrial park development, and integrated resource-based planning for development in industry, agriculture and urban areas (Murray et al., 2017). Considering the impacts of accelerated population growth and rapid urbanisation, coupled with climate change, the urban nexus approach considers spatial planning that reduces new green-field development but promoting the repurposing of already transformed land, including redeveloping degraded infrastructure (Oliveira et al., 2018).

## 4 Discussion

The rapid urbanisation occurring in Gauteng Province is driven by inter-and intra-migration, creating a complex migration-urbanisation relationship. Migrants are attracted by better economic prospects in the province, which is supported by the region’s industrialisation, modernisation, globalisation, marketisation, and good governance (Castelli, 2018), (Gu, 2019). Although the Gauteng Spatial Development Framework acknowledges the need for a balanced polycentric spatial approach in urban planning, the main challenge has been translating the strategy into practice. This study provides a framework that offers pathways that integrate distinct but interlinked sectors, including diverse actors and stakeholders, into a collective engagement to provide integrated and sustainable development.

An urban growth of 30.7% in Gauteng between 2001 and 2011, which increased to 38.2% (an increase of 7.5%) between 2011 and 2015, is a clear indication of the high immigration levels into the province (Bobbins and Culwick, 2015). Between 2011 and 2015, the province recorded a net increase of migrants of about 543,000 people (Bobbins and Culwick, 2015). About 67% of international migration in Gauteng Province is intra-regional, as people come from other countries within southern Africa, and the remaining 23% come from different continents (Castelli, 2018; StatsSA, 2019). As a result, the Gauteng City-Region is now spatially fragmented, scattered, and sprawled, making it difficult to provide essential services and sanitation to all (Chakwizira et al., 2018). However, rapid growth is also contributing to economic development by developing new commercial and industrial nodes, easing pressure from city centres, and reducing concentrated pollution. This has seen the development of one-stop centres such as the Mall of Africa in Johannesburg and Menlyn Maine in Tshwane, with state of the art industrial and office parks, hotels, shops, and restaurants.

Urban sprawl is pronounced in the Gauteng City-Region as evidenced by the number of informal settlements and the increase in the built-up area. The sprouting of informal settlements poses a significant health risk as the unplanned settlements sprout on undesignated areas that do not have any water and sanitation amenities (Owusu-Ansah et al., 2016). The continued emergence of informal settlements is a health time bomb, especially with the frequency in the emergence of novel infectious diseases such as the Covid-19, SARs, and Ebola (WHO, 2020). Moreover, shanty towns have become crime and vice hot spots (Chakwizira et al., 2018). The challenges are exacerbated by the high unemployment rates, which stands at 31% in the province (StatsSA, 2019). Illegal electricity connections are rampant, endangering human life, particularly children (Louw and Bokoro, 2019). The Gauteng City-Region faces the challenge of providing safe and clean water and sanitation, safe and clean energy, and most of the households are food insecure (Mabhaudhi et al., 2019; Nhamo et al., 2020).

### 4.1 Adaptation pathways for urban areas

Responses and strategies to climate change impacts and rapid urbanisation range from autonomous strategies to reactive interventions, and proactive interventions to long-term adaptation strategies, as well as transformational strategies (Mpandeli et al., 2018). Thus, adaptation actions are either reactive and autonomous or anticipatory and planned (proactive adaptation) or transformational. This section details the approaches to either partially, temporarily, or permanently adapt cities to rapid urbanisation and climate change. The latter is the most appropriate as they offer long-lasting solutions.

#### 4.1.1 Reactive or autonomous adaptation

Reactive/autonomous adaptation refers to spontaneous ex-post interventions undertaken after a shock, such as an extreme weather event, or when an urban area is already overcrowded with people (Rahman and Hickey, 2019). They are based on immediate and localised needs but often result in a maladaptive trajectory because of the lack of planning and foresight (Rahman and Hickey, 2019). Thus, reactive adaptation is an endogenous adjustment to a shock and is short-term as it focuses on managing risks and vulnerability in the thick of a shock (Chhetri et al., 2019). An example of a reactive adaptation is temporarily moving people settled in lowlands or wetlands to higher altitudes during flooding. Still, they move back soon after the floods subside without rectifying the underlying problem. These reactive adaptation strategies have become pronounced in Africa due to the increasing frequency and intensity of extreme climatic events (Hunter et al., 2020).

#### 4.1.2 Anticipatory or planned adaptation

On the other hand, anticipatory and planned adaptation actions (proactive) refer to large scale and long-term scenario planning based on goal-specific ex-ante actions (Chhetri et al., 2019). They are focused on longer-term livelihoods security, and are primarily based on scientific evidence, require a large pool of resources, technological know-how, and institutional capacity to implement (Rahman and Hickey, 2019). Anticipatory adaptation involves planned policy and investment decisions that enhance an urban area’s adaptive capacity or any other system that includes building satellite towns further away from city centres to reduce congestion and pressure on infrastructure. These initiatives are bearing fruits in the Gauteng Cities by constructing centres like Sandton City, Menlyn Centre, Mall of Africa, among others. These long-term solutions are easing pressure on established centres as they have office parks and hotels, shops, restaurants, and high-density residential apartments. Such developments repurpose existing transformed land, rather than transforming greenfield areas that provide critical biodiversity areas and ecological infrastructure. In addition to the construction of new centres, several initiatives are taking place to redevelop the urban core, for example, New Town and Maboneng precincts in Johannesburg.

While reactive/autonomous responses are important during short-term interventions, proactive interventions should be pursued, as they enhance long-term resilience sustainability. To ease pressure on already established built-up centres, the Gauteng Provincial Government has established five developmental centres that have distinct industries and different comparative advantages, these include (GPG, 2017):

- Northern Corridor encompasses Tshwane Metropolitan and focuses on automotive industry manufacturing, innovation, research and development, aerospace, and defence industries.
- Central Corridor, which includes Johannesburg, which specialises in the financial sector, the technological nerve centre, and innovation, research, and development.
- Western Corridor, which encompasses mining, tourism, agri-business, and agro-processing.
- Southern Corridor, encompassing the Sedibeng region, has been becoming derelict due to the steel industry’s collapse.
- Eastern Corridor, which includes Ekurhuleni, an international hub of Africa’s largest aerotropolis, with advanced manufacturing and agro-processing capabilities and globally competitive logistics capacity.

Adopting the urban nexus approach is critical for providing integrated pathways to formulate cross-sectoral strategies across the developmental centres applying the urban nexus framework (Fig. 5).

#### 4.1.3 Transformational adaptation

Transformational adaptation refers to an adaptation that completely transforms the fundamental attributes of a system in response to climate change or rapid urbanisation (Few et al., 2017). It is a total transformation and evolution from the norm, to one adapted to prevailing conditions in response to climate change or the high influx of people. It is generally informed by a system that has reached the limits of its ability to maintain its functionality and applies innovative approaches that allow consistent and uninterrupted service delivery (Few et al., 2017). Thus, transformational adaptation involves complete and innovative changes in the whole system to adapt to the new norm after historic approaches have been stretched and become insufficient for prevailing climate risks (Chhetri et al., 2019). Transformational adaptation is different from the other strategies because of its innovation, magnitude, the intensity of change, and its transformative effect on society (Chhetri et al., 2019). Its implementation has seen the emergence of new concepts like Green Village/City and Sustainable City. Adopting transformational adaptation pathways facilitates the efficiency and sustainability of climate solutions. Transformative approaches to urban adaptation, such as urban nexus, recognise the interests, rights, values, and vulnerabilities of historically marginalised groups and communities. They consider these marginalised groups in the decision-making and planning processes and prioritise the equitable distribution of benefits and costs of proposed adaptation interventions (Colloff et al., 2017).

## 5 Recommendations

A resilient city is one with the capacity to withstand and absorb the impact of shocks through resilience or adaptation. It can cope and mitigate the impacts and minimise damage (Kim and Lim, 2016). In the course of a crisis, a resilient city maintains its essential functions and structures and continues offering the same services and amenities without much external intervention (Kim and Lim, 2016). This is possible through anticipatory policy measures and strategies that require investments beforehand, as informed by transformative processes (Chhetri et al., 2019). Such planning determines the preparedness of a city, which also determines the magnitude of damage caused by extreme events (Nhamo et al., 2019). Proper planning provides for proactive interventions, as there is enough lead time to prepare, that shocks may not end as disasters (Nhamo et al., 2019). Thus, the Gauteng cities need to develop the resilience to withstand shocks and absorb the projected massive influx of people. There is a need to design infrastructure in anticipation of future changes and rapid urbanisation. Past and present experiences should be used to inform adaptation strategies. However, such a strategy should provide locally-based solutions as challenges differ from place to place (Mpandeli et al., 2019). The ultimate goal is to ensure that even though urban areas may bend from shocks, they will remain firm and steadfast, and they quickly adapt and rebound to new levels of sustainability with minimum loss or damage (Chhetri et al., 2019). Examples of such cities are Tokyo in Japan and Auckland in New Zealand. Besides the many devastating earthquakes, they are exposed to, they remain resolute and firm with little consequences. With the anticipated high influx of people into the Gauteng City Region, the region should be adapted and be prepared to cope.

Urbanisation should no longer be viewed as a problem, but as an adaptation strategy to the adverse environmental and climatic changes (Chhetri et al., 2019). Not implying that people should be moved into urban areas, but, where necessary to escape from imminent disaster or climate shock, urbanisation should be viewed as an adaptation option. There are many advantages of urbanisation to the receiving city if it possesses a resilient city’s qualities. In any case, decision-makers need to address the drivers of migration in places of origin and put adaptation strategies that would transform the receiving urban area into a resilient city (Chhetri et al., 2019). Migration is considered here as the leading cause of rapid urbanisation. Therefore, addressing the drivers of migration, as well building resilient cities should be a priority in a fast-changing environment, and the following factors should be considered in adaptation strategies for building resilient cities in South Africa and the rest of the region:

- There are great opportunities to benefit from leveraging and enhancing the flow of intra-regional labour migrants into Gauteng cities, as South Africa is faced with the challenge of scarce skills. The free movement of skilled labour in the region should be viewed as an opportunity for regional integration. The USA and the European Union are good examples of regions that are benefiting from such strategies.
- The Southern Africa Development Community (SADC) should oversee intra-regional migration, viewing it as a means of regional integration. As South Africa is a water-scarce country, and its water resources fast deteriorating, the government should consider focusing more on industrial development and prioritising investment in agriculture and energy development in other countries in the region, that have abundant water resources (Nhamo et al., 2018). This will motivate people to stay rather than to migrate. This is only possible through regional integration through multinational arrangements such as SADC.
- Where urbanisation is considered an adaptation strategy, migrants ought to be viewed as resources that are beneficial to themselves and the communities they come from (Satterthwaite, 2017). This positive perspective of urbanisation does not take migration as a burden on destination urban areas or lost brain drain on sending countries. There is a need to tap into migrants’ capabilities and transform them into useful resources, other than placing them into refugee camps. This model has been successful in the European Union (EU) where there is free movement of labour (Schmidt et al., 2018).
- Regional integration enhances coordinated development, creates employment opportunities in countries of origin through resource sharing, as most of the resources are shared through transboundary river basins, but unevenly distributed (Nhamo et al., 2018). For example, investing in untapped agriculture and hydropower generation potential in the Democratic Republic of Congo and Zambia could solve food and energy insecurity in the whole SADC region (Mabhaudhi et al., 2016). However, the slow pace in integration is why the region has chronic challenges of water, energy, and food insecurity, yet is blessed with abundant resources. Regional integration and exploiting own resources would reduce unemployment and poverty, ensure economic development through skills development, and income-generating projects for the youth in migrant-sending countries, reducing migration and rapid urbanisation. The spatial inequalities in development will not be evident where there is regional integration, as there is inclusive and equitable development in all areas.
- The region needs to systematically collect disaggregated data and use it for informed policy decisions that holistically address the drivers of migration and make context-based adaptation decisions. The analysis of such data provides long-term solutions to the challenges posed by migratory patterns such as demographic transitions, and structural transformation in the society as driven by climate change.
- The adoption of an urban nexus approach in urban planning provides more flexible, holistic, practice-oriented, and contextualised solutions that are needed for urban climate change adaptation and mitigation (Nhamo and Ndlela, 2020). Nexus planning provides integrated climate change adaptation and mitigation strategies, identifies priority areas for intervention, enhances synergies, and minimises trade-offs (Mpandeli et al., 2018). Adopting the approach is critical for integrated urban planning as it integrates urban circular economy and scenario planning approaches for inclusive, sustainable development and resilience.
- The Gauteng City-Region should consider redevelopment and repurposing of already transformed land, including city centres, to localise circular economy value chains (González-Sánchez et al., 2020). This approach prevents the greenfield development and continued urban sprawl, which is unsustainable and compromises future generations’ resource base (Dorsey, 2003).
- There are great opportunities for policy and decision-makers to formulate urban strategies that turn cities and towns into sustainable structural transformation and adaptation engines. The 2030 Agenda for Sustainable Development (UNGA, 2015) and the 2016 common African position on urban development provide the basis for understanding migration and urbanisation as a means of promoting regional integration in the SADC region, as the two documents advocate for people to live and work in a healthy, safe and secure environment in any urban area or anywhere in the continent.

## 6 Conclusions

The Gauteng City-Region is witnessing rapid urbanisation, with migration and climate change being identified as the main drivers. Its economic strength and industrial development remain the primary pull factors attracting migrants into the province, and the trend is projected to intensify in the short- to medium-term. A combination of transformative approaches like the circular economy and scenario planning, linked through the WEF nexus, provide solutions to transform urban areas into resilient and sustainable cities of the future. Formulating informed adaptation strategies, through transformative approaches, provides sustainable pathways that systematically integrate short- medium- and long-term planning, hence promoting intergenerational sustainability. Achieving urban sustainability and resilience will transform urban areas into vibrant centres of habitat for humankind and biodiversity, sustainable socio-economic development, and sites for climate action and adaptation. This, while improving human health and well-being and reducing inequalities and vulnerabilities. Urbanisation promotes regional integration and inclusive economic development through the free movement of skilled labour as an adaptation strategy. With most southern countries of the SADC region classified as water-scarce, but industrially developed, the region ought to develop agriculture and energy potential like those from northern countries to create employment in other parts and mitigate migration to metropolitan areas. In an increasingly urbanised world, the development of SSA in the 21st century lies in its cities and towns as the region witnesses the Fourth Industrial Revolution (4IR). Managing urbanisation trends in a holistic and integrated manner will facilitate the simultaneous harnessing of benefits of migration and rapid urbanisation, which include regional integration, social cohesion, and inclusive, equitable, and integrated economic development.

## Figures and Tables

**Table 1:** Increase in the built-up area from 1986 to 2019 in the Gauteng City-Region

Year	Built-up area (km^2^)	Non-built-up area (km^2^)	Proportion of built-up area
1986	1,422	16,748.31	7.3%
1999	2,493	15,677.67	13.7%
2007	3,225	14,945.68	17.8%
2019	3,850	14,320.56	21.2%

**Table 2:** Impact of social and economic development onto the environment (based on critical biodiversity areas in 2011 – Compaan, 2011).

Landuse type	Area in Km^2^	% change of CBA converted
Critical Biodiversity Areas which were converted to agriculture	553.23	-11.7%
Critical Biodiversity Areas which were converted to urban	209.70	-4.4%
Ecological Support Areas which were converted to agriculture	972.44	-29.2%
Ecological Support Areas which were converted to urban	354.76	-10.7%

**Table 3:** Changes in landuse areas due to urban growth in Gauteng Province

Landuse	Area in 2011 (km^2^)	Area in 2018 (km^2^)	Percentage change
Water	731.07	629.67	-13,87
Natural Vegetation	9,439.99	8,456.95	-10,41
Agriculture	4,562.51	4,758.34	4,29
Mine	246.35	224.95	-8,69
Cleared Land	14.94	182.60	1,122,22
Built Up Areas	3,175.41	3,917.76	23,38

***Note***: Negative values represent a decrease in area and a positive increase in area

**Table 4:** Vehicular pollutants and CO_2_ in Gauteng compared to national emissions (tons/yr.) in %.

	NO_2_	SO_2_	CO	PM_10_	NMVOC	Benzene	Lead	CO_2_
National	251,390	6952	1,241,295	13,646	184,161	319	0.53	54,258,926
Gauteng(%)	29.44	24.94	35.46	24.28	34.71	32.92	28.30	29.88
